# Calculation of vessel pulse wave velocities in retinal vein segments within the optic disc centre

**DOI:** 10.1038/s41598-024-79995-8

**Published:** 2024-11-26

**Authors:** A. Vukmirovic, W. H. Morgan, D. Obreschkow, A. Abdul-Rahman, D. Y. Yu, A. Mehnert

**Affiliations:** 1 Lions Eye Institute, Centre for Ophthalmology and Visual Science, University of Western Australia, Crawley, Australia; 2 International Centre for Radio Astronomy Research (ICRAR), M468, University of Western Australia, 35 Stirling Hwy, Crawley, WA 6009 Australia; 3 Department of Ophthalmology, Counties Manukau DHB, Auckland, New Zealand; 4 International Space Centre, 35 Stirling Hwy, Crawley, WA 6009 Australia

**Keywords:** Biomedical engineering, Medical research

## Abstract

The carotid-femoral pulse wave velocity (PWV) method is used clinically to determine degrees of stiffness and other indices of disease. It is believed PWV measurement in retinal vessels may allow early detection of diseases. In this paper we present a new non-invasive method for estimating PWVs in retinal vein segments close to the optic disc centre, based on the measurement of blood column pulsation in retinal veins (reflective of vessel wall pulsation), using modified photoplethysmography (PPG). An optic disc (OD) PPG video is acquired spanning three cardiac cycles for a fixed ophthalmodynamometric force. The green colour channel frames are extracted, cropped and aligned. A harmonic regression model is fitted to each pixel intensity time series along the vein centreline from the centre to the periphery of the OD. The phase of the first harmonic is plotted against centreline distance. A least squares line is fitted between the first local maximum phase and first local minimum phase and its slope used to compute PWV. Five left eye inferior hemi-retinal veins from five healthy subjects were analysed. Velocities were calculated for several induced intraocular pressures ranging from a mean baseline of 14 mmHg (SD 5) to 56 mmHg in steps of approximately 5 mmHg. The median PWV over all pressure steps and subjects was 20.77 mm/s (IQR 29.27). The experimental results show that pulse wave propagation direction was opposite to flow in this initial venous segment.

**Supplementary Information:**

The online version contains supplementary material available at 10.1038/s41598-024-79995-8.

**Authors**:

## Introduction

The retina provides a non-invasive view of microvascular circulation and the central nervous system^1^. Imaging of the retina can be used to diagnose retinal diseases and potentially systemic and neural diseases by characterising pressure and flow waves in the microvasculature^1^. However, the direct measurement of these waves isn’t currently feasible^2^. In this paper we propose an indirect approach based on the analysis of video of visible pulsation in retinal veins acquired using a modified photoplethysmography (PPG) technique^3–5^. In particular we propose a method for estimating the vessel pulse wave velocity (PWV) based on the phase-shift (time displacement) of the pulse wave measured at multiple sites along a retinal vein segment close to the optic disc centre.

A wave is a phenomenon where energy alternates between kinetic and potential forms creating physical disruptions in the medium it propagates through^6^. The cardiac cycle gives rise to both pressure and flow waves. Pressure waves represent variations in blood pressure that propagate through vessels. Flow waves represent variations in blood flow through a vessel. The vessel pulse wave represents the visible variation in vessel wall displacement arising from both of these^6^. Pressure waves often travel at speeds faster than the bulk flow speeds of the medium it passes through^6^. It’s also important to distinguish between PWV and wave speed which is akin to instantaneous pulse wave velocity at a particular location^2^. The speed of a pressure pulse wave is related to the elasticity, radius and thickness of the medium it traverses^7^. If the medium is elastic, isotropic and linear with minimal mechanical disturbances the wave speed, *c*, of the pressure pulse wave is given by the Moens-Korteweg equation:^7^.

1 $$\:c=\:\sqrt{\frac{Eh}{2\rho R}}$$

where *E* is the elastic modulus of the vessel, *h* is vessel wall thickness, $\rho\:$ is the density of blood, and *R* is the internal radius of the vessel. This equation is commonly used to calculate the wave speed in arteries^7^. Equation (1) shows that ${c}^{2}$ is directly proportional to the elastic modulus of the medium and thus is a measure of the wall stiffness the wave propagates along^7^. In a recent study, Rahman et al. used a video ophthalmodynamometer to estimate a vessel pulse wave velocity of 22.2 mm/s in the retinal vein of a single subject based on retinal vascular pulse wave parameters together with manual measurements of diameter changes from the induced pulsation^8^. In our experience, these diameter changes are difficult to detect in contrast to the pulsation-induced intensity changes (related to blood column thickness). In another recent study, Laloy-Borgna et al. used laser doppler holography to measure retinal pulse wave velocities via retinal blood flow^9^. They calculated PWVs in both arteries and veins to be between 1 and 10 mm/s^9^. However, laser doppler holography is challenging to perform, time consuming and requires a skilled operator. In addition, laser doppler holography systems are complex and expensive (requiring laboratory conditions)^10^. In this paper we present a new method for estimating pulse wave velocities in retinal veins using a modified photoplethysmographic (PPG) technique. The approach is non-invasive, well-tolerated, quick to perform, and can be performed by a trained health care worker. We demonstrate the methodology using five eyes from five healthy subjects.

## Methods

In this section we present the new method for estimating PWV in retinal veins, and also an experiment where the method was used to estimate PWV in five left eye inferior veins from five healthy subjects, for several induced intraocular pressures ranging from a mean baseline of 14 mmHg (SD$\:5$) to 56 mmHg (Note: mean and SD have been rounded to the nearest integer). PWV measurements were performed at varying intraocular pressures because we did not wish to assume constancy of pulse wave velocity in the face of possible transmural pressure changes, i.e., variation in difference between the venous intraluminal pressure and its external chamber pressure^4^.

### New method for estimating PWV in retinal veins

**Fig. 1:**
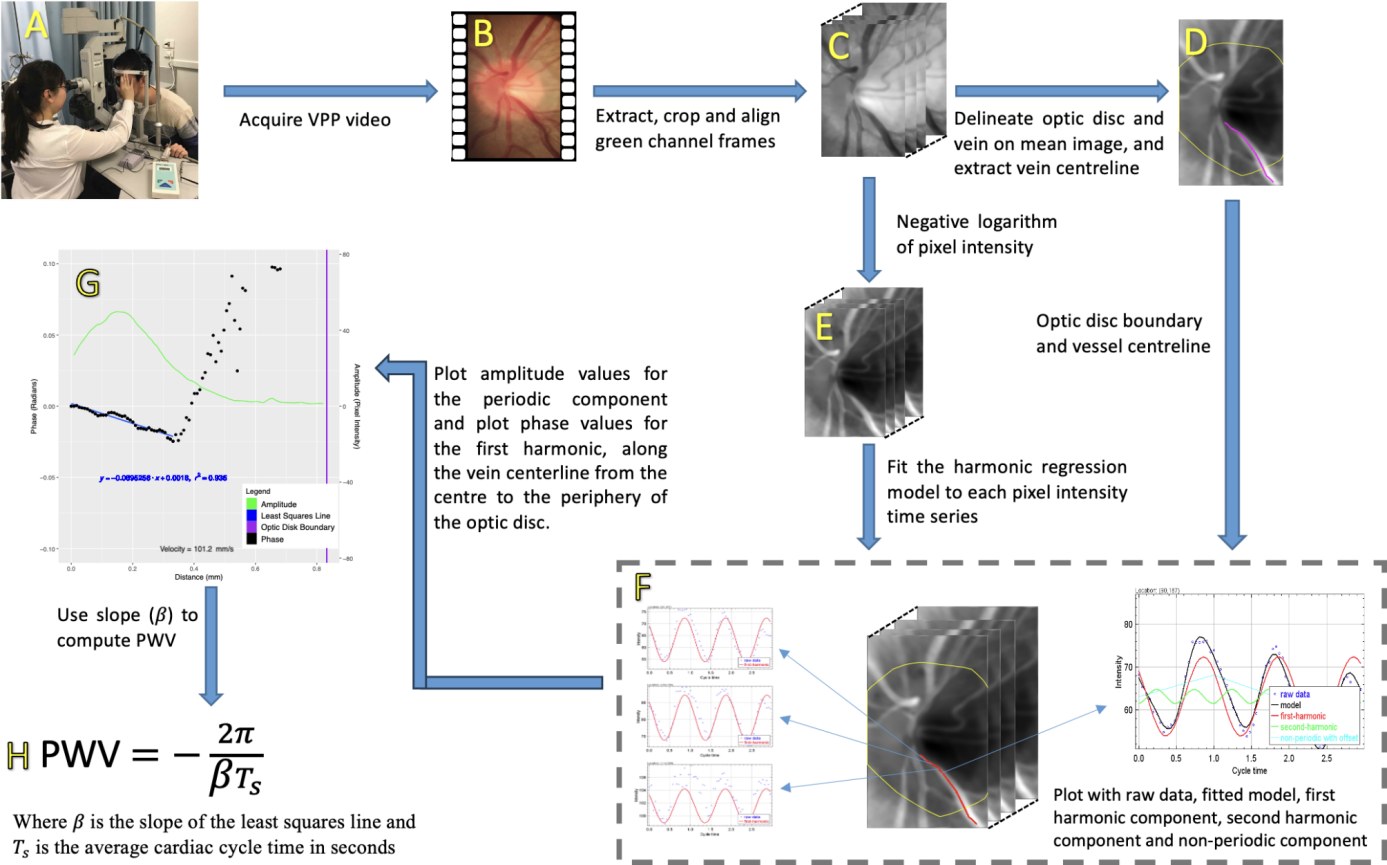
Proposed method for estimating venous pulse wave velocity. (**A**) Acquisition setup showing the patient seated at the video ophthalmodynamometer with the pulse oximeter affixed to their left index finger. (**B**) Acquired video frames over 3 cardiac cycles. (**C**) Extracted green channel images (shown in greyscale) after replacement of blurry video frames and alignment to the sharpest frame. (**D**) Manual segmentation/delineation of the optic disc boundary and the vessel of interest. The centerline is then given by the skeleton (medial axis) of the vessel mask. (**E**) Negative log transform of the pixel intensities in (**C**) so that intensity is related to blood column thickness and hence vessel diameter (Beer-Lambert law). (**F**) The harmonic regression model is fitted to each pixel time series in (**E**). The fitted model, first harmonic component, second harmonic component, and non-periodic component are shown for one of the centerline pixels. The first harmonic of the periodic component is shown for 3 centerline pixels. (**G**) Phase of the first harmonic and the amplitude of the periodic component both plotted against distance along the vessel centerline (from the point closest to the optic disc centre to the periphery of the optic disc). (**H**) Computation of PWV from the slope of the least squares line fitted to the distance-phase points.

The new method comprises the following steps, which are illustrated in Fig. 1.

***Step 1: Acquire PPG video***.

The PPG video is acquired using the technique previously described in Morgan  (2015)^3^, Morgan (2016)^4^ and Morgan (2022)^5^. Briefly, the technique utilizes a Meditron ophthalmodynamometer (Meditron, Volklingen, Germany), which essentially consists of a three-mirror Goldmann lens connected to a ring force transducer capable of continuous force output. During video-photography, the subject wears a pulse oximeter affixed to their right index finger, and the audible oximeter ‘beep’ is recorded simultaneously with the video. The subject is seated in front of a slit lamp camera (Carl Zeiss, Germany), while video recordings are captured at a rate of 25 frames per second, containing on average 70 frames using a Canon 5D mark III (Canon Corp, Japan)(Fig. 1A-B). Each frame is a 24-bit RGB image of size 1920 × 1080 pixels. A minimum of three consecutive cardiac cycles are recorded^3–5^.

***Step 2: Extract green channel frames and align all frames to the sharpest frame***.

The green channel is extracted from each video frame (chosen because a haemoglobin absorption peak coincides with an isobestic point for oxy- and deoxyhaemoglobin at 550 nm). The set of frames is then partitioned into 3 cardiac cycles. The sharpest frame is identified. This involves applying a Laplacian of Gaussian filter to each image frame (the filter is based on the second-order derivatives of the image and tends to highlight image edges)^11^, computing the standard deviation of the pixel intensities in each filtered frame, and selecting the frame with the largest standard deviation. Blurry frames (any frame with a sharpness value < 75% of the value for the sharpest frame) are replaced by the nearest non-blurry frame. Each frame is then aligned to the sharpest frame. Alignment is performed in a sequential fashion, beginning with translation-only registration based on local key features derived from the scale-invariant feature transform (SIFT)^12^, followed by rigid registration, and finally affine registration (Fig. 1C). Post alignment, the frame size is cropped to the bounding box of the optic disc (typically 200 × 300 pixels).

***Step 3: Manually delineate (segment) the optic disc and vein of interest***,*** and extract centreline***.

The average (pixel-wise intensity average) of all the frames is used to define a region-of-interest (ROI) corresponding to the optic disc, and another corresponding to the vein for which the vessel PWV is to be estimated. The single pixel thick centreline of the vein is given by the skeleton (medial axis) of the binary mask of the vein ROI (Fig. 1D). The size of each pixel in mm is determined from an optical coherence tomography (OCT) scan of the optic disc of the same eye. In particular, the OCT vendor’s software is used to measure the vertical diameter of the optic disc in mm, and this is equated to the number of pixels in the vertical diameter of the previously defined optic disc ROI.

***Step 4: Transform intensity values***.

Following alignment of each pixel the negative logarithm of the green colour channel pixel intensity values in arbitrary units (Au) are computed. Using principles of the Beer Lambert law, each value is related to blood column thickness in the axial dimension so that variation over time relates to vessel wall axial diameter variation (Fig. 1E)^4,5,13^.

***Step 5: Fit a harmonic regression model to each pixel intensity time series***.

The following harmonic regression model, which we have detailed in our previous work^8,14,15^, is fitted to each pixel time series using generalised least squares (maximising the restricted log-likelihood):

2 $$\:y\left(t\right)={f}_{\text{p}}(t)+{f}_{\text{n}\text{p}}(t)+{\epsilon}_{t}$$

where ${f}_{\text{p}}$ is the periodic component, ${f}_{\text{n}\text{p}}$ is the non-periodic component (linear spline with knots at the start and end of each cardiac cycle), and ${\epsilon}_{t}$ is a first-order autoregressive error component^8^. The time $t$ is the fraction of the cardiac cycle rather than time in seconds. The periodic component is modelled as the Fourier series expansion, in sine-cosine form, up to the second harmonic, i.e.,

3 $$\:{f}_{p}\left(t\right)={a}_{0}+{\sum\:}_{n=1}^{2}{a}_{n}\text{cos}\left(2\pi nt\right)+{b}_{n}\text{sin}\left(2\pi nt\right)$$

where ${a}_{i}$ and ${b}_{i}$ are real coefficients, and $t$ represents time as a fraction of the cardiac cycle rather than seconds (the sine-cosine expansion is amenable to fitting using generalised least squares). The fitted model parameters at a given pixel are used to quantify the vessel pulsation at that location^4^. Figure 1F shows an example of the model fitted at a single pixel location, and additionally shows the periodic and non-periodic components. The difference between the maximum and minimum values of the periodic component is hereinafter called the harmonic regression wave amplitude (HRWA). Figure 1F also shows the first harmonic of the periodic component for 3 different locations along the centerline of the vein.

***Step 6: Extract phase and compute PWV***.

The Fourier series expansion in Eq. (3) can be equivalently written in the following amplitude-phase form:

4 $$\:{f}_{p}\left(t\right)={A}_{0}+{\sum\:}_{n=1}^{2}{A}_{n}\text{sin}\left(2\pi nt+{\varphi}_{n}\right)$$

where ${A}_{0}={a}_{0}$, ${A}_{n}=\sqrt{{a}_{n}^{2}+{b}_{n}^{2}}$ is the *magnitude* of the *n*-th harmonic and $\:{\varphi}_{n}={\text{tan}}^{-1}\left({b}_{n}/{a}_{n}\right)$ is the *phase* angle of the *n*-th harmonic.

**Fig. 2:**
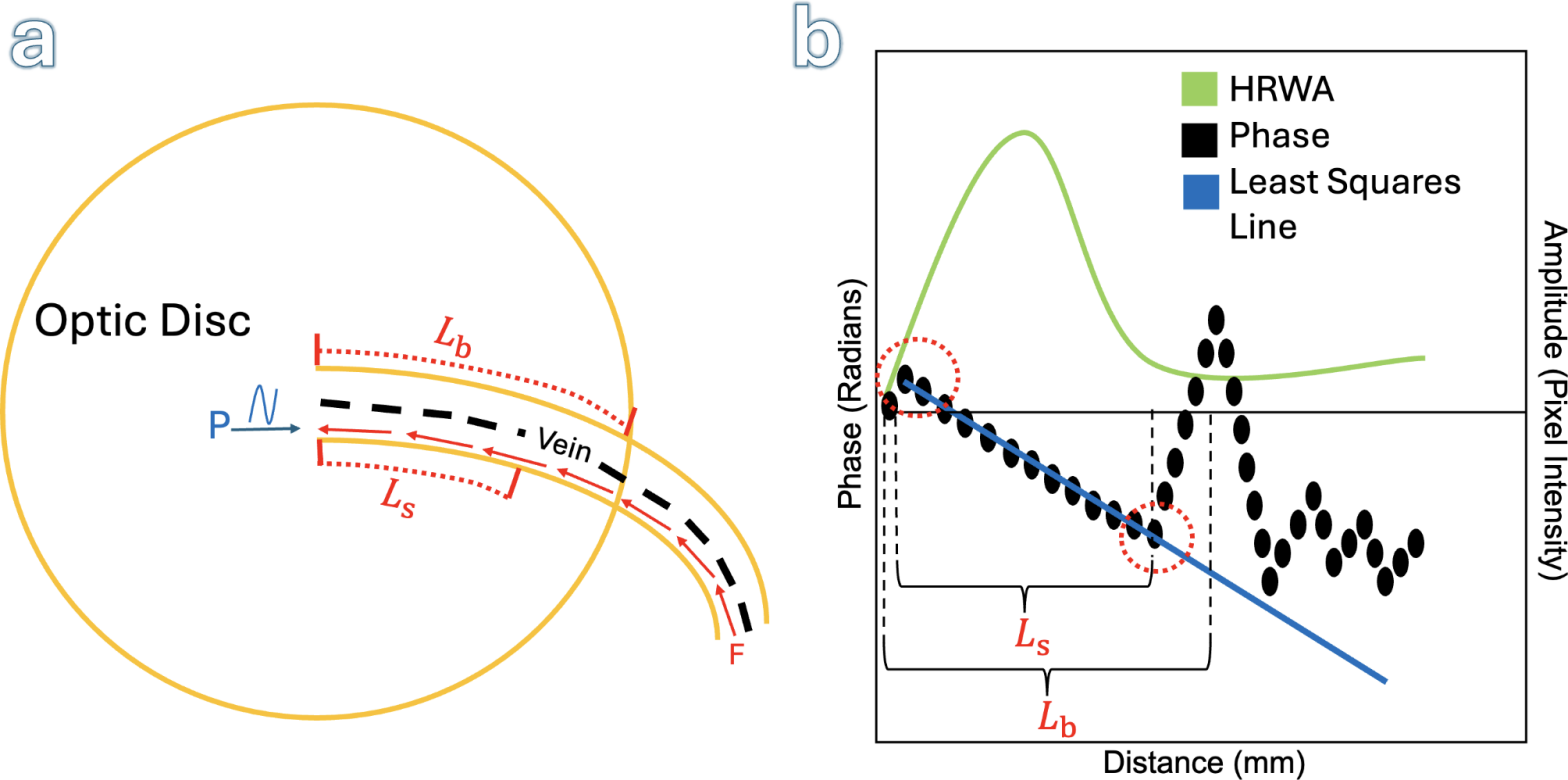
Schematic showing how the segment of the vein centreline used to compute the PWV is selected. (**a**) Shows the optic disc (OD) and selected vein. The vein centreline is shown as a dashed black line. The arrow at P indicates the direction of the measured venous pulse wave. The arrows originating from F indicate the direction of blood flow within the vein. ${L}_{\text{boundary}}$ (${L}_{\text{b}}$) is the length of the centreline path from the origin (point closest to the centre of the OD) to the OD boundary. The segment of the path, of length ${L}_{\text{segment}}\left({L}_{\text{s}}\right)$, used to compute the PWV is determined from (**b**), which shows a plot of the phase of the first harmonic of the fitted harmonic regression model and the HRWA against distance along the vessel centreline. The segment corresponds to the closed interval, of width ${L}_{\text{segment}}$, defined by the first local phase maximum and first local phase minimum from the origin (both indicated by dotted red circles). The slope of the least squares line fitted to the distance-phase points in this interval is then used to compute the PWV (see text for details).

The phase of the first harmonic, ${\varphi}_{1}$, is computed for each centreline pixel within the optic disc. The sequence of centreline pixels defines a path. The distance of each pixel along this path is computed relative to the centreline pixel closest to the centre of the optic disc. These geodesic distances are calibrated relative to the height of the optic disc in mm. The phase values of the pixels along the path are normalized so that the first pixel on the path has a value of zero (by subtracting the initial value from all values). The phase of the first harmonic and the amplitude of the periodic component are both plotted against distance. A least squares line is then fitted to the distance-phase points in the closed interval defined by the first local maximum closest to the origin, and subsequent first local minimum closest to the origin (see Fig. 2). Notably, the HRWA attains its maximum value in this interval. The slope of the least squares line is used to compute the PWV:

5 $$\:c=\:-\frac{2\pi\:}{\:\beta\:{T}_{s}}$$

where $\beta\:$ is the slope of the least squares line, and ${T}_{\text{s}}$ is the average cardiac cycle time in seconds (average of the 3 recorded cycles). The derivation is shown in Appendix A.

#### Implementation

Steps 2–6 of the method were implemented using custom scripts written in FIJI^16^ and R^17^ (version 4.2.2). At step 5, the implementation also computes *R*-squared adjusted as a measure of the goodness-of-fit of the fitted model^18^. At step 6, the implementation also includes loess smoothing (local polynomial regression) of the distance-phase values (loess function in R, with span 0.5) to facilitate automatic identification of the local maximum and local minimum used to define the segment of the vein centreline used for PWV estimation.

### Experiment: PWV estimation for 5 healthy cases

The proposed method was used to estimate the PWV in five left eye inferior veins from five healthy subjects, for several induced intraocular pressures ranging from a mean baseline intraocular pressure of 14 mmHg (SD $5$) to 56 mm Hg in steps of approximately 5 mmHg. The subjects (four male) were between the ages of 26 and 33 years. The use of human subjects for the PPG measurements was approved by Belberry Human Research Ethics Committee (permit number 2015-11-756-A-2), in accordance with the declaration of Helsinki and in compliance with National Health and Medical Research Council guidelines for clinical trials. All measurements were performed according to relevant guidelines and regulations and informed consent was obtained from all participants. Subjects in Fig. 1 have provided informed consent for publication of identifying information/images in an online open-access publication. All measurements were obtained using standard clinical protocols for equipment cleaning and best practice following Lions Eye Institute guidelines. In each case, the subject had a prior optical coherence tomography (OCT) scan of the optic disc (Heidelberg Engineering, Heidelberg, Germany), from which the vertical diameter of the optic disc in mm was measured. This in turn was used to calibrate the size of a pixel in mm in the acquired PPG video frames.

### Data availability

The datasets used in the study are available from the corresponding author upon reasonable request.

### Statistical analysis

All statistical analyses were performed in R using the $\alpha\:=0.05$ level of significance. The package nlme^19^ was used for fitting linear mixed effects models. The mean, standard deviation, and range were computed across all subjects for each of the following (see Fig. 2): length of the vein centreline path from the origin to the OD boundary (${L}_{\text{boundary}}$) and the length of the centreline path (${L}_{\text{segment}}$) used to compute the PWV. The PWV results have been summarised using medians and interquartile ranges (IQRs) to avoid reporting negative PWVs for mean plus or minus SD. A linear mixed effects model was fitted with PWV as the response variable; age, sex, induced intraocular pressure, maximum HRWA, ${L}_{\text{segment}}$ as explanatory variables (fixed effects); and subject as the random effect. A Q-Q plot of the residuals and a plot of the residuals versus predicted values were used to check that model assumptions were satisfied. Backward elimination was used to iteratively remove uninformative explanatory variables; at each step the variable with the highest p-value was identified and if its p-value was greater than the level of significance it was removed^20^. The intraclass correlation coefficient for the final model was found by dividing the random effect variance by the total model variance.

## Results

Where measurements were made on an integer scale (e.g., pixel counts), means, standard deviations, medians and IQRs are reported to one decimal place. For all other measurements they are reported to two decimal places. All p-values are reported to three decimal places.

**Fig. 3:**
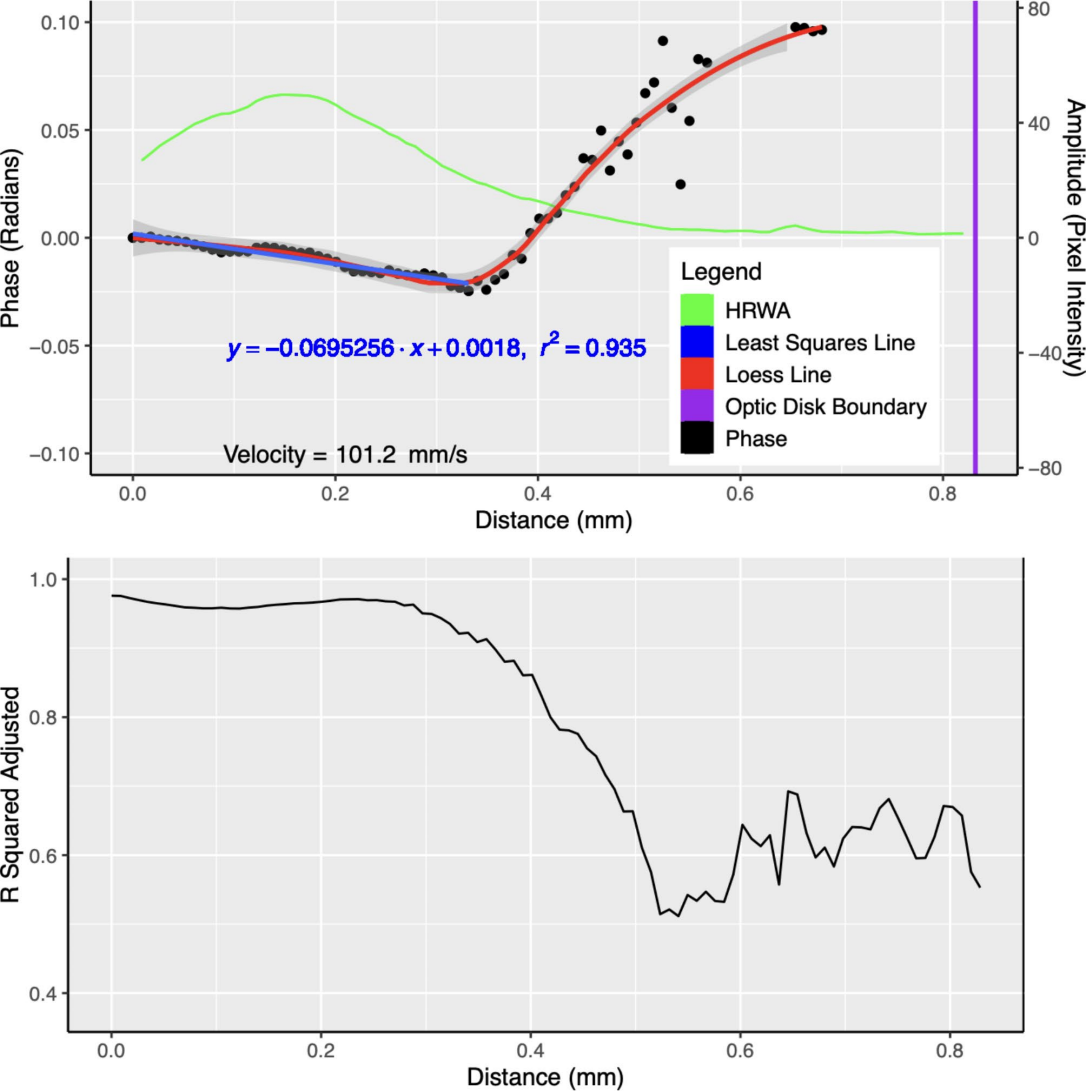
Top: Plot of the phase of the first harmonic of the fitted harmonic regression model, and the amplitude of the periodic component (HRWA), versus distance along the vessel centre line (origin is the point closest to the centre of the optic disc) for subject D. The least squares line used to compute the PWV is shown in blue while the loess curve is shown in red. Phase values are shown as black dots (see text for details). Bottom: Plot of *R*-squared adjusted versus Distance (mm). R-squared adjusted is a measure of the goodness of fit of the model^18^.

**Fig. 4:**
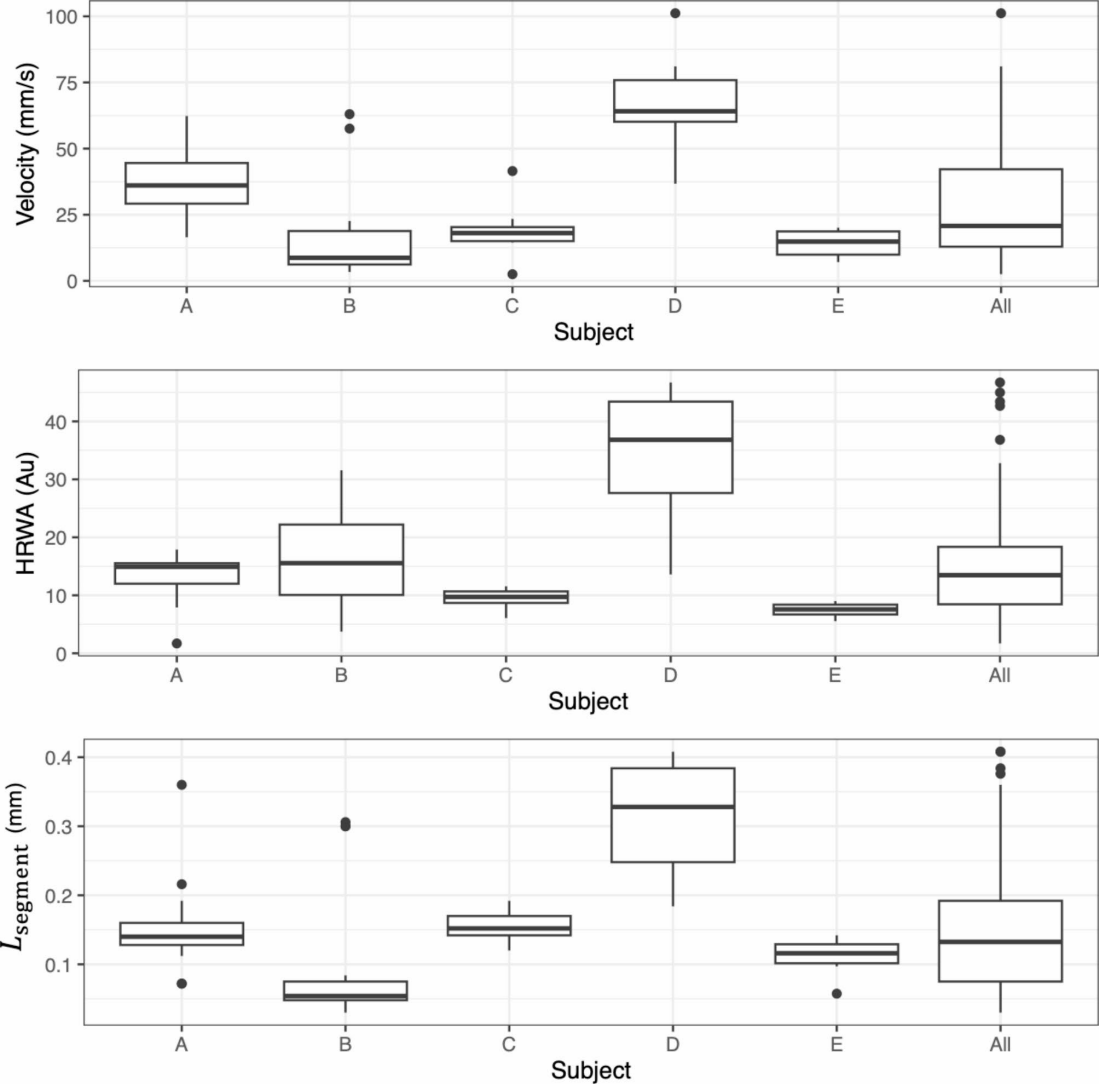
Side-by-side boxplots of PWV, HRWA, and ${L}_{\text{segment}}$for all subjects.

Figure 3 shows the plot of phase, HRWA and *R*-squared adjusted against distance; the loess curve through the distance-phase points; and the fitted least squares line to the selected distance-phase points, for subject D. For the 5 subjects across all pressures, the mean number of vein centreline pixels, from the centre of the OD to the boundary, was 77.2 (SD $24.4$). This equates to a measurement of phase an average of every 0.01 mm (SD $0.00$). The mean ${L}_{\text{boundary}}$ was 0.57 mm (SD $0.18$), ranging from 0.33 mm to 0.83 mm. The mean ${L}_{\text{segment}}$ was 0.16 mm (SD $0.10$), ranging from 0.03 to 0.41 mm, corresponding to a mean of 23.7 (SD $13.7$) phase measurements per vein. The median speed (PWV magnitude) across all five subjects and induced intraocular pressure steps was 20.77 mm/s (IQR 29.27), with speeds ranging from 2.51 mm/s to 101.16 mm/s. The PWV across all induced intraocular pressures for each respective subject (Fig. 4) were: Subject A 36.07 mm/s (IQR 15.36), Subject B 8.71 mm/s (IQR 12.66), Subject C 18.07 mm/s (IQR 5.27), Subject D 64.12 mm/s (IQR 15.73), Subject E 14.84 mm/s (IQR 8.78). The median of the medians for all subjects was 18.07 mm/s.

From the fitted linear mixed effects model obtained using backwards elimination a statistically significant relationship was identified between PWV and two of the explanatory variables, maximum HRWA (*p* < 0.001, c = 1.23 velocity (mm/s) / HRWA) and ${L}_{\text{segment}}$ (*p* < 0.001, c = 111 velocity (mm/s) / least squares line (mm)). No statistical significance was found amongst the other explanatory variables of age, sex and induced intraocular pressure in the model. The intraclass correlation coefficient computed from the model was 0.66, indicating a moderate correlation between PWVs for the same individual over a range of induced intraocular pressures.

## Discussion

Our method allows the measurement of a pulse wave as it propagates along a retinal vein by tracking the phase of the wave along the vessel length. Our results demonstrate that in the segment of the vein close to the centre of the optic disc, and coinciding with the maximum pulsation, there is a vessel pulse wave travelling towards the optic disc boundary i.e., in the direction opposite to blood flow, with a median speed of 20.77 mm/s (IQR 29.27) over all subjects and induced intraocular pressures. The intraclass correlation coefficient of 0.66 suggests there is relative consistency of the PWV estimates within an individual. Variation in IOP did not appear to be associated with PWV. Previous studies based upon pig servo-null pressure measurements have found that transmural pressures appear to be relatively constant in the face of varying intraocular pressure measurements^21^. These previous findings appear to agree with our finding of a moderate intraclass correlation coefficient (0.66) between PWVs within the same individual. However, our results also seem to suggest a significant variation in PWVs between individuals. There may be interesting properties within the retinal vein walls, which account for this variation, but at present, we are unable to identify these properties.

The Moens-Korteweg equation requires a medium that is elastic, isotropic and linear with minimal mechanical disturbances^7^. It is unlikely that retinal veins meet this requirement and as such the Moens-Korteweg equation may not be applicable for use in retinal veins. This means that while the equation describes a specific relationship between wave speed and elasticity of a medium, such as vessel wall, this may not hold true for retinal veins. The measurement of pulse wave velocities in veins may allow the analysis of venous wall stiffness in a similar manner to the analysis of arterial stiffness via arterial pulse waves and thereby may have value as a predictor of diseases such as hypertension, diabetes mellitus and stroke^22^. In cases where retinal venous pulsation is not visible to the observer, as is the case for many patients with microvascular dysfunction, the applicability of our methodology for measuring PWVs remains an open question. We intend to explore this in future work.

The PWV values measured using our method characterise the speed and direction of the vessel pulse wave in the portion of the retinal hemi vein in which maximum pulsation occurs. Whilst several studies have reported venous PWVs, it is difficult to relate these to our measurements for several reasons including differences in anatomical location, differences in measurement techniques, and differences in physiological meaning (e.g., measurements of blood flow rather than the speed of propagation of the vessel pulse wave). Nippa et al. measured pulsatile blood flow changes at a known distance apart between the subclavian and femoral veins using two transcutaneous ultrasonic flow detectors to estimate a mean PWV in ten subjects of 1.15 m/s (SD 0.33)^23^. Ermini et al. induced pressure pulses in the femoral vein through rapid compression of the foot and then measured the time taken for those pulse waves to travel a known distance using doppler ultrasound^24^. Ermini et al. estimated the pulse wave velocity in a subject in the supine position as 1.78 m/s (SD 0.06)^24^. George et al. developed a dual ultrasound system capable of simultaneously acquiring vessel wall echoes from two proximally spaced points along the internal jugular vein and found pulse wave velocities between 1.54 and 1.82 m/s in ten subjects^25^. Recently there have been studies of pulse wave velocities in retinal veins by Laloy-Borgna et al. using laser doppler holography and by Rahman et al. using modified photoplethysmography who found PWVs in retinal veins between 1 and 10 mm/s and 22.2 mm/s respectively^8,9^. The reported venous PWVs measurements in anatomy other than the retina seem to be comparable but differ by orders of magnitude to the results reported in this paper. However, the reported retinal venous PWVs are of the same order of magnitude as the results reported in this paper.

Previous studies have explored pulse wave velocities in retinal arteries as well with a wide range of results that differ in orders of magnitude between differing papers and methods. However, the venous PWV measurements presented in this paper can only be approximately compared to PWVs that have been calculated in retinal arteries and capillaries from previous literature because of methodological differences used to measure PWVs in retinal veins, arteries and capillaries. Kotliar et al. in 2013 found pulse wave velocities in retinal arteries of 0.442 mm/s in normotensive subjects utilising the dynamic vessel analyser (DVA) method^26^. DVA allows the measurement of changes in diameter of retinal arteries along an artery and thereby allowing the calculation of a phase shift between two points along the selected artery^26^. PWV is then calculated by dividing distance over the phase shift between two points along the selected artery^26^.

In 2015 Spahr et al. estimated PWV in retinal arteries to be 620 mm/s (SD 50) using phase-sensitive full-field swept-source optical coherence tomography^27^. Spahr’s technique uses a two point method, a distal and a proximal point along the artery and allows the changes in the axial expansion of vessel walls to be measured in nanometers^27^. Spahr et al. then used the time delay between two pulse signals to calculate vessel pulse wave velocity^27^. In 2018, Li et al. estimated the PWV in retinal arteries in a young healthy subject between 20 and 30 mm/s using spectral domain optical coherence tomography^28^. Li et al. used phase resolved doppler OCT to derive the pulse shape in the form of pulsatile blood flow at two sites along a retinal artery and then estimated the pulsatile blood flow wave velocity from the transit time it took the wave to traverse the two sites. In 2021, Bedggood and Metha found the average PWV in retinal capillaries of three subjects as 6.4 mm/s (SD 0.5)^29^. Bedggood and Metha used a high spatiotemporal resolution adaptive optics system that allowed the continuous tracking in space and time of individual cells within each capillary tube^29^. Laloy-Borgna et al. also found retinal arterial pulse wave velocities between 1 and 10 mm/s using laser doppler holography^9^.

The fitted linear mixed model indicates that as HRWA and ${L}_{\text{segment}}$ (length of centreline path, used to compute the PWV) increases, PWV increases. Greater pulsation in veins, and therefore higher recorded HRWA, is related to greater transmural pressure difference variation^4^. This finding seems consistent with the Bramwell-Hill equation which describes an association between an increase in the pressure pulse wave velocity and an increased change in pressure^30^. Additionally, ${L}_{\text{segment}}$ was different for each case. A greater ${L}_{\text{segment}}$ was found to be related to a greater PWV. This finding indicates an unusual aspect of the nature of the vessel pulse wave. The pulse wave directly near the origin of the vein (< 0.05 mm) initially appears to travel at a slower velocity before increasing its velocity as it moves along the vessel. Visible pulsation in the vein is seen within the initial section of the vein closest to the centre of the optic disc. This finding may also relate to the area of maximum pulsation and as such greater transmural pressure changes. In this situation the pulse wave speed increases as it starts to enter the location of greater pulsation.

Compared to previous methodologies, this new methodology allows the phase analysis at each pixel along a retinal vein. The tracking of the wave’s phase at each pixel in this manner allows an examination of the vessel pulse wave as it propagates along a retinal vein and thereby enabling the calculation of speed and direction of that wave to obtain the vessel PWV. A peculiar observation from our results is that after an initial linear downward trend (coinciding with the maximum pulsation amplitude), the phase exhibits an upwards trajectory. The reason for this is unknown. However, it does suggest a wave may be coming from the opposite direction perhaps through reflection or another mechanism.

There are several limitations with our methodology. The accuracy of the venous PWV measurements can be impacted by unevenly distributed pulsations around the optic disc. Unevenly distributed pulsations may arise due to anatomical wall dimension variations and potentially intraluminal pressure gradient variations. The signal to noise ratio (SNR) of the estimated PWV depends on the length of the vein segment used to derive the estimate. The shorter this length, the lower the SNR. Our results indicate that the trajectory of phase of the first harmonic along the vessel centreline path from the OD centre to the OD boundary, is initially linear with a downward slope (coinciding with the location of the point of maximum pulsation amplitude HRWA). They also indicate the existence of a subsequent turning point where the trajectory takes an upward turn. The reason for this is unknown, although opens the possibility of some variation in pulse wave transmission distal to the initial venous segment close to the optic disc centre. The small amount of data (5 subjects) used to fit our linear mixed model may have limited its ability to detect a statistically significant relationship between PWV and IOP. Our methodology measures PWV using the first harmonic. Previous studies in the dog ascending aorta have found that PWVs vary greatly with frequency^31^. In these studies the first harmonic was found to overestimate the pulse wave velocity when compared to the analysis of higher order harmonics^31^. Additionally, the efficacy of using PWV in pulsating retinal veins as a proxy measure of retinal venous stiffness has yet to be proven. There may be a use in certain diseases that cause change in vessel wall elasticity like diabetes.

Our method demonstrates the possibility of continuous measurement of phase components of a pulse wave at each pixel along a retinal vein. This allows the possibility to calculate retinal pulse wave velocities which may be useful in acting as predictors of disease.

## Electronic supplementary material

Below is the link to the electronic supplementary material.

Supplementary Material 1

## Data Availability

The datasets used during the study are available from the corresponding author upon reasonable request.
