# Supplementary material for: Calculation of vessel pulse wave velocities in retinal vein segments within the optic disc centre
Source: Sci Rep. 2024 Nov 26;14:29404. doi: 10.1038/s41598-024-79995-8 (PMC11599586; doi:10.1038/s41598-024-79995-8)
Supplement: Supplementary file 1 — Supplementary Material 1 [file 41598_2024_79995_MOESM1_ESM.docx]

Appendix A: Derivation of the expression for PWV

From equation 4, the first harmonic is given by

$A\sin(2\pi t+\phi)$ Equation (A1)

where$t$ is the fraction of the cardiac cycle rather than time in seconds and $\varphi$ is phase.


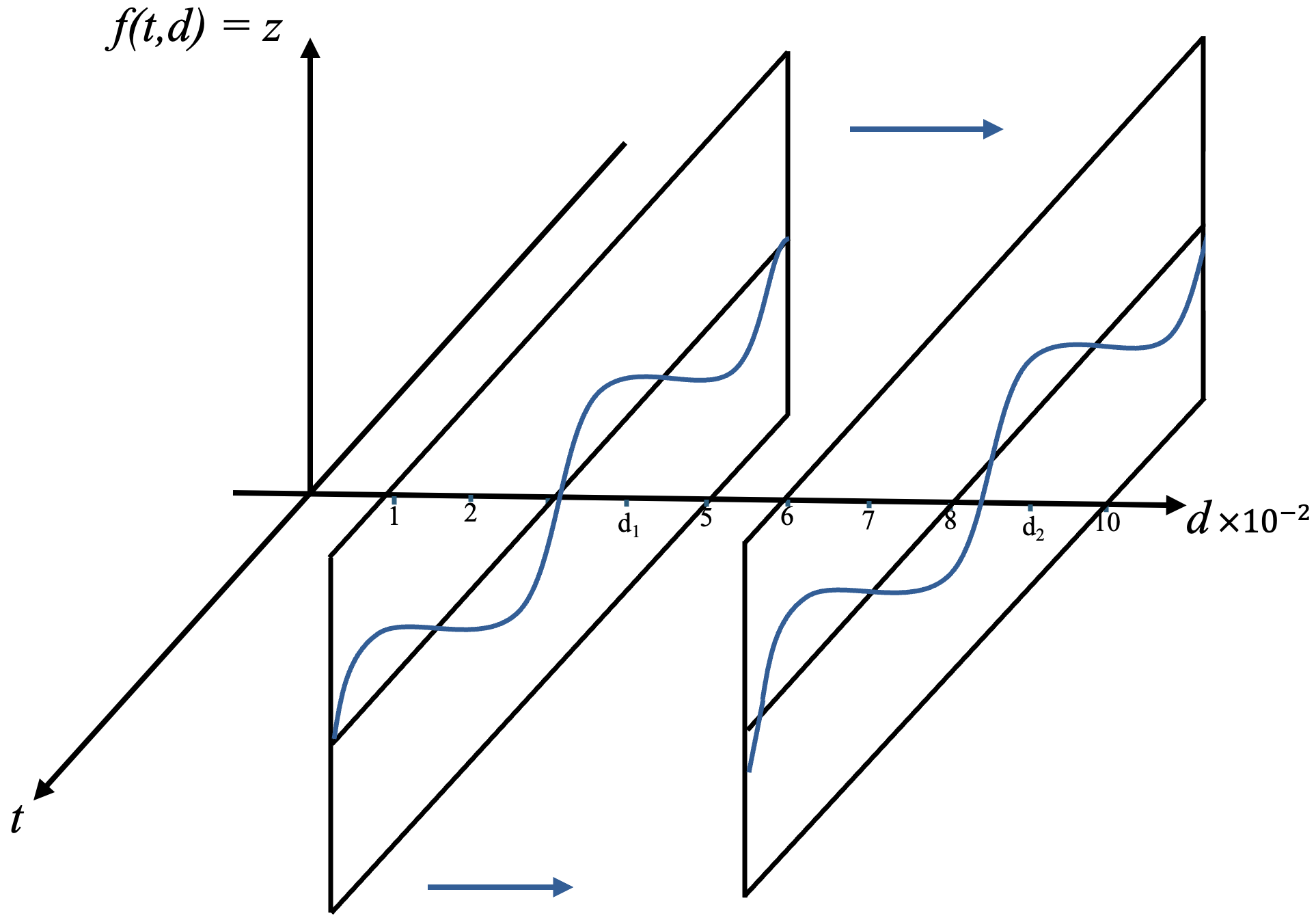


Figure A1 is a schematic showing the first harmonic of the fitted harmonic regression model at distance $d_{1}=0.0314$ mm and $d_{2}=0.0832$ mm along the vein centreline. The set of first harmonic waveforms for all distances along the centreline then defines a surface $z=f(t,d)$ where $d$ is the distance from the origin and *t* is the fraction of the cardiac cycle time.

Let $d_{1}$ and $d_{2}$ be two points along the vein centreline ($d$ axis in Figure A1) such that $d_{1}<d_{2}$. Let $\mathrm{Asin} \left( 2\pi t+\phi_{1} \right)$ denote the first harmonic of the fitted harmonic regression model at $d_{1}$, and $\mathrm{Asin} \left( 2\pi t+\phi_{2} \right)$ be the first harmonic of the fitted model at $d_{2}$. The first harmonic at $d_{2}$ should be the same as that at $d_{1}$ at an earlier time $t-\tau$ where $\tau>0$. This can be written:

$\mathrm{Asin} \left( 2\pi\left( t-\tau\right)+\phi_{1} \right)=\mathrm{Asin} \left( 2\pi t+\phi_{2} \right)$ Step 1

$2\pi(t-\tau)+ \phi_{1}= 2\pi t +\phi_{2}$ Step 2

$-2\pi\tau= \phi_{2}-\phi_{1}$ Step 3

$\tau= -\left( \phi_{2}-\phi_{1} \right)/2\pi$ Step 4

Let $T_{s}$ denote the average cardiac cycle time in seconds (average of the 3 recorded cycles). Then $\tau\times T_{s}$ represents the time in seconds it took the wave to travel between the two points.

Thus, the velocity of the wave $z=f(t,d)$ in the $d$ axis direction is the PWV and is given by:

$c= -\frac{2\pi\left( d_{2}-d_{1} \right)}{T_{s}\left( \phi_{2}-\phi_{1} \right)}$ Equation (A2)

If we replace $\left( d_{2}-d_{1} \right)/\left( \phi_{2}-\phi_{1} \right)$with the reciprocal of the slope of the fitted least squares line from Step 6 we obtain Equation 5:

$$c= -\frac{2\pi}{\beta T_{s}}$$
